# Supplementary material for: LC-MS/MS-Based Fungicide Accumulation Assay to Demonstrate Efflux Activity in the Wheat Pathogen Zymoseptoria tritici
Source: Microorganisms. 2022 Jul 25;10(8):1494. doi: 10.3390/microorganisms10081494 (PMC9331226; doi:10.3390/microorganisms10081494)
Supplement: Supplementary file 1 [file microorganisms-10-01494-s001.zip › microorganisms-1733084-supplementary.pdf]

Table S1. EC_{50} values observed with different fungicides during *in vitro* growth of *Zymoseptoria tritici* in 96-well plates. Values were obtained as described in Experimental procedures, in a preliminary single assay. N. D. = not determined.

Strain	EC_{50} (µg/mL)				
	Carboxin	Boscalid	Tolnaftate	Fludioxonil	Fenpiclonil
IPO323	2.407	0.838	1.836	1.424	0.469
IPO323Δ*MFS1* T1	N. D.	0.358	0.358	0.494	0.027
IPO323Δ*MFS1* T2	0.003	0.051	0.358	0.098	0.752
IPO323*MFS1*$^{\text{MDR-TypeI}}$ 6.18	1.962	20.615	N. D.	1.981	0.945
IPO323*MFS1*$^{\text{MDR-TypeI}}$ 6.20	0.991	12.091	9.086	0.836	0.851

Table S2. Evolution of the relative intracellular percentage of boscalid in *Z. tritici* during seven days of growth in 24-well plates in YSS medium.

Day	Relative intracellular percentage of boscalid			
	IPO323	IPO323Δ*mfs1**	IPO323*MFS1*$^{MDR\text{-}TypeI}$*	SE31
0	1.83 ± 0.21	0.48 ± 0.04	0.89 ± 0.004	1.50 ± 0.18
1	1.93 ± 0.45	1.54 ± 0.16	2.48 ± 1.24	1.51 ± 0.05
2	1.38 ± 0.21	2.17 ± 0.45	1.10 ± 0.03	1.62 ± 0.01
3	5.53 ± 0.44	9.04 ± 0.76	1.28 ± 0.38	2.96 ± 0.20
4	15.65 ± 1.40	35.29 ± 0.87	2.33 ± 0.11	2.89 ± 0.12
7	16.05 ± 2.08	24.28 ± 2.32	2.14 ± 0.36	18.28 ± 0.12

*indicated values are means of n=2 biological replicates of two independent mutants per genotype

Table S3. EC_{50} values (µg/mL) of tested *Z. tritici* strains towards boscalid. Values were obtained as described in Experimental procedures (N = 2-10 independent assays).

Strain	Day	EC_{50} (µg/mL)
IPO323	3	0.519 ± 0.045
	4	0.527 ± 0.031
	7	1.553 ± 0.327
IPO323Δ*mfs1**	3	0.185 ± 0.011
	4	0.231 ± 0.052
	7	0.27 ± 0.053
IPO323*MFS1*$^{\text{MDR-TypeI}}$*	3	2.098 ± 0.247
	4	3.819 ± 0.586
	7	6.2 ± 0.391
SE31	3	0.498 ± 0.025
	4	0.663 ± 0.030
	7	0.87 ± 0.033

*indicated values are means of n=2 biological replicates of two independent mutants per genotype
